# Genome Sequence of the Mucoromycotina Fungus *Umbelopsis isabellina*, an Effective Producer of Lipids

**DOI:** 10.1128/genomeA.00071-14

**Published:** 2014-02-27

**Authors:** Itaru Takeda, Koichi Tamano, Noriko Yamane, Tomoko Ishii, Ai Miura, Myco Umemura, Goro Terai, Scott E. Baker, Hideaki Koike, Masayuki Machida

**Affiliations:** a Bioproduction Research Institute, National Institute of Advanced Industrial Science and Technology (AIST), Tsukuba, Ibaraki, Japan; b Department of Biotechnology and Life Science, Tokyo University of Agriculture and Technology, Tokyo, Japan; c Bioproduction Research Institute, National Institute of Advanced Industrial Science and Technology (AIST), Sapporo, Hokkaido, Japan; d Computational Biology Research Center (CBRC), National Institute of Advanced Industrial Science and Technology (AIST), Tokyo, Japan; e Environmental Molecular Sciences Laboratory, Pacific Northwest National Laboratory, Richland, Washington, USA

## Abstract

*Umbelopsis isabellina* is a fungus in the subdivision Mucoromycotina, many members of which have been shown to be oleaginous and have become important organisms for producing oil because of their high level of intracellular lipid accumulation from various feedstocks. The genome sequence of *U. isabellina* NBRC 7884 was determined and annotated, and this information might provide insights into the oleaginous properties of this fungus.

## GENOME ANNOUNCEMENT

*Umbelopsis isabellina* is a Mucoromycotina fungus belonging to the order Mucorales, which includes the genera *Rhizopus*, *Mucor*, *Cunninghamella*, etc. Interestingly, *U. isabellina* was formerly classified in the order Mortierellales, but a molecular phylogenetic analysis has shown that *Umbelopsis* species belong in the Mucorales order (1).

Many fungi in the subdivision Mucoromycotina, including the well-known pathogen *Rhizopus oryzae*, have been shown to be oleaginous (2, 3). Both *U. isabellina* and *Cunninghamella echinulata* accumulate lipids to more than 40% of their biomass (4). Because of their high levels of intracellular triacylglyceride accumulation, versatility in nutrient utilization, and high growth rate, Mucoromycotina fungi are becoming increasingly important as potential producers of biofuels and high-value chemicals (5, 6). The genome sequences of at least two Murocomycotina fungi have been published—*R. oryzae* (7) and the Mortierellales fungus *Mortierella alpina* (3). Moreover, other genome sequences are currently accessible through the Joint Genome Institute (JGI) Mycocosm Genome Portal (8), including *Mucor circinelloides*, *Umbelopsis rammanianna*, and *Phycomyces blakesleeanum*. However, the numbers of Mucormycotina genome sequences are still much lower than those of other lineages of Dikarya fungi. The comparative study of the Mucoromycotina fungi could be useful for determining the genetic factors related to oleaginous characteristics because they share oleaginous characteristics. However, only small regions of microsynteny have been observed among the genome sequences currently available (our unpublished data).

The fungal strain used in this study, *U. isabellina* NBRC 7884, was obtained from the Japanese Biological Resource Center, NITE (http://www.nbrc.nite.go.jp/). Whole-genome sequencing was performed using a mate-paired library with an insert size of approximately 3 kb and a read length of 55 bases with the SOLiD 5500xl system (Life Technologies). The total short reads were filtered according to quality (9), resulting in approximately 200-fold coverage (85,662,706 reads), and assembled by SOLiD De Novo Accessory Tools 2.0 (Life Technologies), which contains the Velvet assembler (10), with a k-mer size of 37. The assembly generated 84 scaffolds (≥1 kb) with an *N*_50_ value of 554,975 bp and a maximum length of 1,873,367 bp. The assembled genome of 22,588,838 bp was covered by 84 scaffolds, including 53 scaffolds of >100 kb and 4 scaffolds of >1 Mb, with a G+C content of 41.79%.

A total of 9,081 protein-coding genes were predicted, based on the homologies to known genes and the statistical features of the genes, by applying a combination of gene-finding software tools (11). Functional annotation of the genes was predicted by a homology search (BLASTp) against an NCBI-nr protein database. A total of 6,911 of 9,081 protein-coding genes (76.1%) were homologous to sequences in the annotated genes of *U. ramanniana* sequenced by the Department of Energy (DOE) Joint Genome Institute (8) (BLASTp *E* value, 1e−5; sequence length, ≤20% difference; and sequence identity, ≥25%).

### Nucleotide sequence accession numbers.

This whole-genome shotgun project has been deposited at DDBJ/EMBL/GenBank under the accession numbers BAVE01000001 through BAVE01000084. The version described in this paper is the first version.

## References

[1] Voigt K Wöstemeyer J 2001 Phylogeny and origin of 82 zygomycetes from all 54 genera of the Mucorales and Mortierellales based on combined analysis of actin and translation elongation factor EF-1alpha genes. Gene 270:113–120. 10.1016/S0378-1119(01)00464-4 11404008

[2] Vorapreeda T Thammarongtham C Cheevadhanarak S Laoteng K 2012 Alternative routes of acetyl-CoA synthesis identified by comparative genomic analysis: involvement in the lipid production of oleaginous yeast and fungi. Microbiology 158:217–228. 10.1099/mic.0.051946-0 22016567

[3] Wang L Chen W Feng Y Ren Y Gu Z Chen H Wang H Thomas MJ Zhang B Berquin IM Li Y Wu J Zhang H Song Y Liu X Norris JS Wang S Du P Shen J Wang N Yang Y Wang W Feng L Ratledge C Zhang H Chen YQ 2011 Genome characterization of the oleaginous fungus mortierella alpina. PLoS One 6:e28319. 10.1371/journal.pone.0028319 22174787 PMC3234268

[4] Fakas S Papanikolaou S Batsos A Galiotou-Panayotou M Mallouchos A Aggelis G 2009 Evaluating renewable carbon sources as substrates for single cell oil production by *Cunninghamella echinulata* and *Mortierella isabellina*. Biomass Bioenerg. 33:573–580. 10.1016/j.biombioe.2008.09.006

[5] Rossi M Amaretti A Raimondi S Leonardi A 2011 Getting lipids biodiesel from oleaginous fungi, p 71–92 *In* Stoytcheva M (ed), Biodiesel—feedstocks and processing technologies. InTech, Rijeka, Croatia

[6] Ratledge C 2004 Fatty acid biosynthesis in microorganisms being used for single cell oil production. Biochimie 86:807–815. 10.1016/j.biochi.2004.09.017 15589690

[7] Ma LJ Ibrahim AS Skory C Grabherr MG Burger G Butler M Elias M Idnurm A Lang BF Sone T Abe A Calvo SE Corrochano LM Engels R Fu J Hansberg W Kim JM Kodira CD Koehrsen MJ Liu B Miranda-Saavedra D O’Leary S Ortiz-Castellanos L Poulter R Rodriguez-Romero J Ruiz-Herrera J Shen YQ Zeng Q Galagan J Birren BW Cuomo CA Wickes BL 2009 Genomic analysis of the basal lineage fungus *Rhizopus oryzae* reveals a whole-genome duplication. PLoS Genet. 5:e1000549. 10.1371/journal.pgen.1000549 19578406 PMC2699053

[8] Grigoriev IV Nordberg H Shabalov I Aerts A Cantor M Goodstein D Kuo A Minovitsky S Nikitin R Ohm RA Otillar R Poliakov A Ratnere I Riley R Smirnova T Rokhsar D Dubchak I 2012 The genome portal of the Department of Energy Joint Genome Institute. Nucleic Acids Res. 40:D26–D32. 10.1093/nar/gkr947 22110030 PMC3245080

[9] Umemura M Koyama Y Takeda I Hagiwara H Ikegami T Koike H Machida M 2013 Fine de novo sequencing of a fungal genome using only SOLiD short read data: verification on *Aspergillus oryzae* RIB40. PLoS One 8:e63673. 10.1371/journal.pone.0063673 23667655 PMC3646829

[10] Zerbino DR Birney E 2008 Velvet: algorithms for de novo short read assembly using de Bruijn graphs. Genome Res. 18:821–829. 10.1101/gr.074492.107 18349386 PMC2336801

[11] Machida M Asai K Sano M Tanaka T Kumagai T Terai G Kusumoto K Arima T Akita O Kashiwagi Y Abe K Gomi K Horiuchi H Kitamoto K Kobayashi T Takeuchi M Denning DW Galagan JE Nierman WC Yu J Archer DB Bennett JW Bhatnagar D Cleveland TE Fedorova ND Gotoh O Horikawa H Hosoyama A Ichinomiya M Igarashi R Iwashita K Juvvadi PR Kato M Kato Y Kin T Kokubun A Maeda H Maeyama N Maruyama J Nagasaki H Nakajima T Oda K Okada K Paulsen I Sakamoto K Sawano T Takahashi M Takase K Terabayashi Y Wortman JR Yamada O Yamagata Y Anazawa H Hata Y Koide Y Komori T Koyama Y Minetoki T Suharnan S Tanaka A Isono K Kuhara S Ogasawara N Kikuchi H 2005 Genome sequencing and analysis of *Aspergillus oryzae*. Nature 438:1157–1161. 10.1038/nature04300 16372010

